# Increased number of deaths during a chikungunya epidemic in Pernambuco, Brazil

**DOI:** 10.1590/0074-02760170124

**Published:** 2017-09

**Authors:** Carlos Alexandre Antunes de Brito, Maria Glória Teixeira

**Affiliations:** 1 Universidade Federal de Pernambuco, Recife, PE, Brasil; 2 Universidade Federal da Bahia, Instituto de Saúde Coletiva, Salvador, BA, Brasil

**Keywords:** chikungunya, arbovirus, increased death, Pernambuco

## Abstract

In early 2016, it was suspected that there were more deaths in Pernambuco than in previous years during an epidemic of chikungunya. This study tested whether there was an increased number of deaths and, if so, whether this increase could be related to a chikungunya epidemic. Indeed, there was an increase of 4235 deaths in 2016 compared to the average of the four previous years, and the highest differences were found during the peak period of the epidemic. It was evident that not all of these deaths could be attributed to complications of chikungunya. However, considering the temporal overlap, some of these deaths may have been caused by the aggravation of pre-existing comorbidities or complications caused directly by chikungunya virus infection.

In March 2016, physicians and nurses reported an increased number of deaths of patients with a clinical presentation compatible with chikungunya in different cities in the state of Pernambuco, Brazil. However, prior to November 2016, only 54 deaths had been officially recorded as resulting from this arbovirus, which may represent under-notification (Collucci 2016, MS 2016).

As several cities in Pernambuco were experiencing chikungunya epidemics (SESPE 2016), a hypothesis was raised that chikungunya was causing an increased number of deaths not identified by physicians as being the result of viral infection because of limited knowledge about the potential complications of this emerging disease in Brazil. For many of these deaths, it is possible that only the secondary cause was registered, such as pulmonary or heart disease, without reference to or a diagnosis of chikungunya, or that the deaths were reported as being suspected of being due to other viral or bacterial infections.

Because data on mortality were unavailable, and considering that most of the deaths occurred in a hospital environment, data from the System of Hospital Information/SIH (MS 2016) could represent an adequate indirect source for analysing this problem. Therefore, we compared the number of hospital deaths that occurred from January to November 2016 to the number of deaths from same period of the four previous years in Pernambuco. We assessed the absolute number of excess deaths, calculating mainly the percentage increase compared with the average of the previous years [Excess deaths per month (EDM): excess deaths in 2016 compared with the average of the four previous years in absolute number and in percentage]. We also assessed whether there was an increase in 2016 in the absolute number and percentage compared with the equivalent months with the highest number of deaths occurred from 2012 to 2015 [Excess deaths compared with the month higher numbers of deaths (EDMHD): excess of deaths in 2016 compared with the months greatest number of deaths from 2012 to 2015].

This analysis showed that there was an excess of 4235 deaths in Pernambuco state in 2016 when compared with the average of the previous four years (Figure), with the highest differences focused in the period from January to April, which corresponded to the peak period of the chikungunya epidemic. In Pernambuco, in the four first months of 2016, there were 2919 deaths, which was compared to the average of previous years: January (increase of 33%), February (48%), March (66.1%), and April (40%). If we compared the absolute number of deaths that occurred in the first four months of 2016 with the months that registered the greatest number of deaths in any year from 2012 to 2015, it remains an important difference, which overall indicated an excess of 2061 deaths in the four months (Figure).

**Figure:**
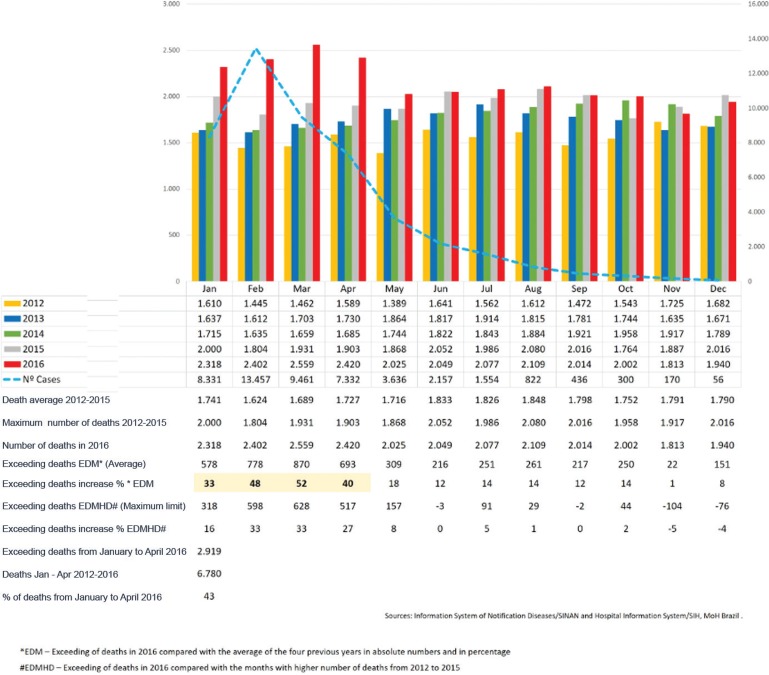
Number of reported cases of chikungunya virus infection (in 2016) and number of hospital deaths from all causes according to year and month in Pernambuco, Brazil, from 2012 to 2016.

It is evident that not all of these additional deaths could be attributed to complications of chikungunya virus infections. However, given the temporal overlap of the highest proportion of excess deaths during the peak period of the chikungunya epidemic, it is possible that some of these deaths may be due to aggravation of pre-existing comorbidities associated with chikungunya virus infection or be consequences of neurological complications directly caused by this virus. On Reunion Island, a total of 121 (60%) fatalities were considered to be deaths caused directly by infection, while others were indirect and were mainly consequences of the decompensation of previous comorbidities (the most common affected age group was the elderly). Another 123 cases were classified as severe and exhibited the following main reasons for hospitalisation: respiratory failure (19 cases), cardiovascular decompensation (18 cases), meningoencephalitis (16 cases), severe hepatitis (11 cases), major cutaneous lesions (10 cases), renal insufficiency (7 cases), and other causes (Renault et al. 2007).

Notably, the increased lethality caused by chikungunya above what has been recorded in official records has already been reported by other authors in other countries (Beesoon et al. 2008, Mavalankar et al. 2008).

The data presented in this study indicate that it is necessary to conduct further research using structured retrospective approaches to evaluate the causes of these excess deaths (Figueiró et al. 2011) to identify any potential clinical-epidemiological associations with chikungunya virus. Other strategies may be implemented to prepare for future epidemics based on studies that were able to detect cases of arbovirus-related deaths not reported by the health system but that were diagnosed by autopsy services (Cavalcanti et al. 2016). Alternatively, they may inform an expansion of the surveillance system to increase the sensitivity of case detection (Renault et al. 2007), which could include reports of doctors outside of the sentinel network, visits to hospitals, data from health insurance funds and self-reports by the population. Finally, we believe that these case studies are critical for the elaboration of specific protocols about alarm signals and special clinical management approaches for severe cases of chikungunya, mainly in patients with comorbidities, to reduce the morbidity and lethality associated with this disease.
